# The genome sequence of the cut surfclam, *Spisula subtruncata *(da Costa, 1778)

**DOI:** 10.12688/wellcomeopenres.22286.1

**Published:** 2024-05-21

**Authors:** Patrick Adkins, Anna Holmes, Andrew Mackie, Teresa Darbyshire, Marine Biological Association Genome Acquisition Lab, Darwin Tree of Life Barcoding collective, Wellcome Sanger Institute Tree of Life Management, Samples and Laboratory team, Wellcome Sanger Institute Scientific Operations: Sequencing Operations, Wellcome Sanger Institute Tree of Life Core Informatics team, Tree of Life Core Informatics collective, Darwin Tree of Life Consortium

**Affiliations:** 1 The Marine Biological Association, Plymouth, England, UK; 2 Amgueddfa Cymru, Cardiff, Wales, UK

**Keywords:** Spisula subtruncata, cut surfclam, genome sequence, chromosomal, Venerida

## Abstract

We present a genome assembly from a specimen of *Spisula subtruncata* (the cut surfclam; Mollusca; Bivalvia; Venerida; Mactridae). The genome sequence is 930.8 megabases in span. Most of the assembly is scaffolded into 19 chromosomal pseudomolecules. The mitochondrial genome has also been assembled and is 19.64 kilobases in length.

## Species taxonomy

Eukaryota; Opisthokonta; Metazoa; Eumetazoa; Bilateria; Protostomia; Spiralia; Lophotrochozoa; Mollusca; Bivalvia; Autobranchia; Heteroconchia; Euheterodonta; Imparidentia; Neoheterodontei; Venerida; Mactroidea; Mactridae; *Spisula*; *Spisula subtruncata* (da Costa, 1778) (NCBI:txid31202).

## Background

Surf clams (Mactridae) are commonly eaten worldwide and are an important fisheries resource ( Degraer *et al.*, 2007; Fahy *et al.*, 2003; Kuykendall *et al.*, 2017). *Spisula subtruncata* is one of three British *Spisula* species and is found in silty muddy sand from the low intertidal and shallow shelf depths around the UK. *S. subtruncata* is a filter feeder, preferring silty or muddy sands and has a northeast Atlantic distribution which extends from Norway and south to Spain continuing into the Mediterranean ( GBIF Secretariat, 2024).

*Spisula subtruncata* has a thick, solid shell but the outline is variable, and two forms exist. One is very fat, squat with large, swollen umbones and a sculpture of heavy concentric lines, the anterior dorsal margin is shorter than the posterior dorsal and the posterior margin is subtruncate. The other form is more elongated, has finer concentric lines but also has a subtruncate posterior margin. In both forms the pallial sinus is short, moderately curved and points towards the anterior margin. The shell is white or cream and is covered with a thin, pale brown periostracum covering it, which wears off in patches ( Degraer *et al.*, 2007).

*S. subtruncata* can be confused with a non-native species that has been discovered in the UK – *Mulinia lateralis*. This American species was first discovered in Europe in 2017 and has recently been discovered in the UK ( Holmes *et al.*, 2023). The non-native has a distinct radial ridge on the posterior margin, enabling a distinction between the two species.

Here we present a chromosomal-level whole genome sequence for *Spisula subtruncata*, based on a specimen from Plymouth Sound, Devon, UK.

## Genome sequence report

**Figure 1.**
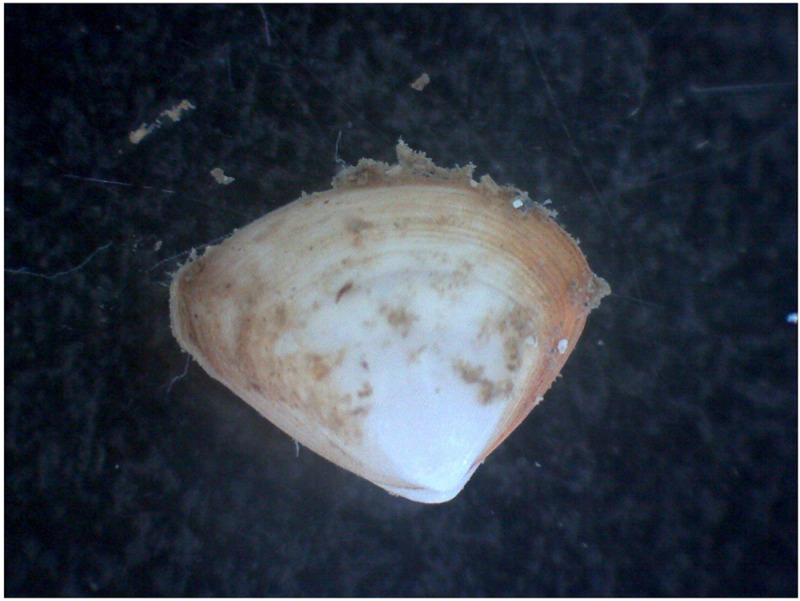
Photograph of the *Spisula subtruncata* (xbSpiSubt1) specimen used for genome sequencing.

The genome was sequenced from a specimen of *Spisula subtruncata* ( Figure 1) collected from Drakes Island East, Plymouth Sound, Devon, UK (50.35, –4.15). A total of 31-fold coverage in Pacific Biosciences single-molecule HiFi long reads was generated. Primary assembly contigs were scaffolded with chromosome conformation Hi-C data. Manual assembly curation corrected 98 missing joins or mis-joins and removed 73 haplotypic duplications, reducing the assembly length by 3.71% and the scaffold number by 29.63%, and decreasing the scaffold N50 by 2.99%.

**Figure 2.**
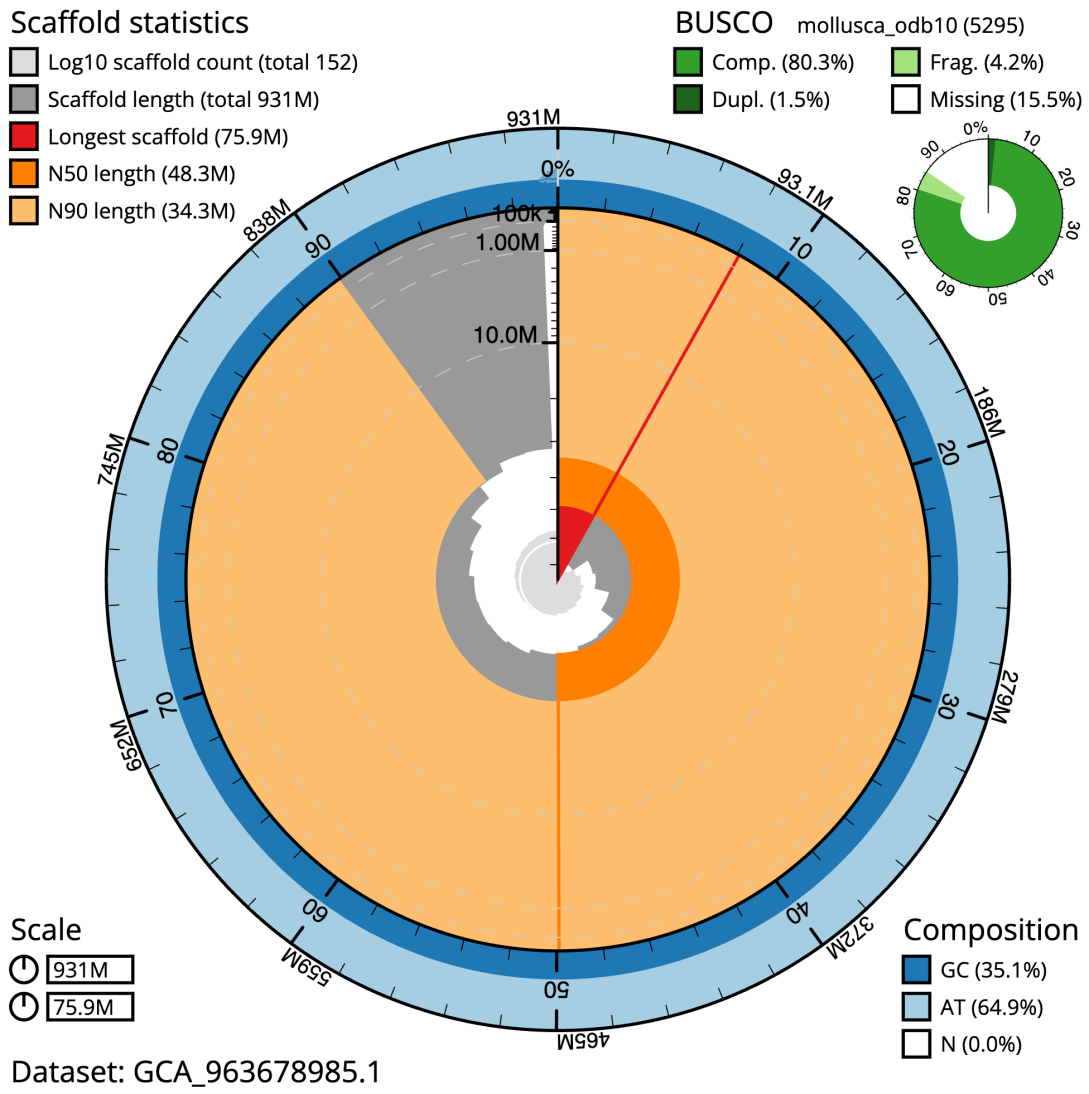
Genome assembly of *Spisula subtruncata*, xbSpiSubt1.1: metrics. The BlobToolKit snail plot shows N50 metrics and BUSCO gene completeness. The main plot is divided into 1,000 size-ordered bins around the circumference with each bin representing 0.1% of the 930,862,144 bp assembly. The distribution of scaffold lengths is shown in dark grey with the plot radius scaled to the longest scaffold present in the assembly (75,865,956 bp, shown in red). Orange and pale-orange arcs show the N50 and N90 scaffold lengths (48,288,179 and 34,259,483 bp), respectively. The pale grey spiral shows the cumulative scaffold count on a log scale with white scale lines showing successive orders of magnitude. The blue and pale-blue area around the outside of the plot shows the distribution of GC, AT and N percentages in the same bins as the inner plot. A summary of complete, fragmented, duplicated and missing BUSCO genes in the mollusca_odb10 set is shown in the top right. An interactive version of this figure is available at https://blobtoolkit.genomehubs.org/view/Spisula_subtruncata/dataset/GCA_963678985.1/snail.

**Figure 3.**
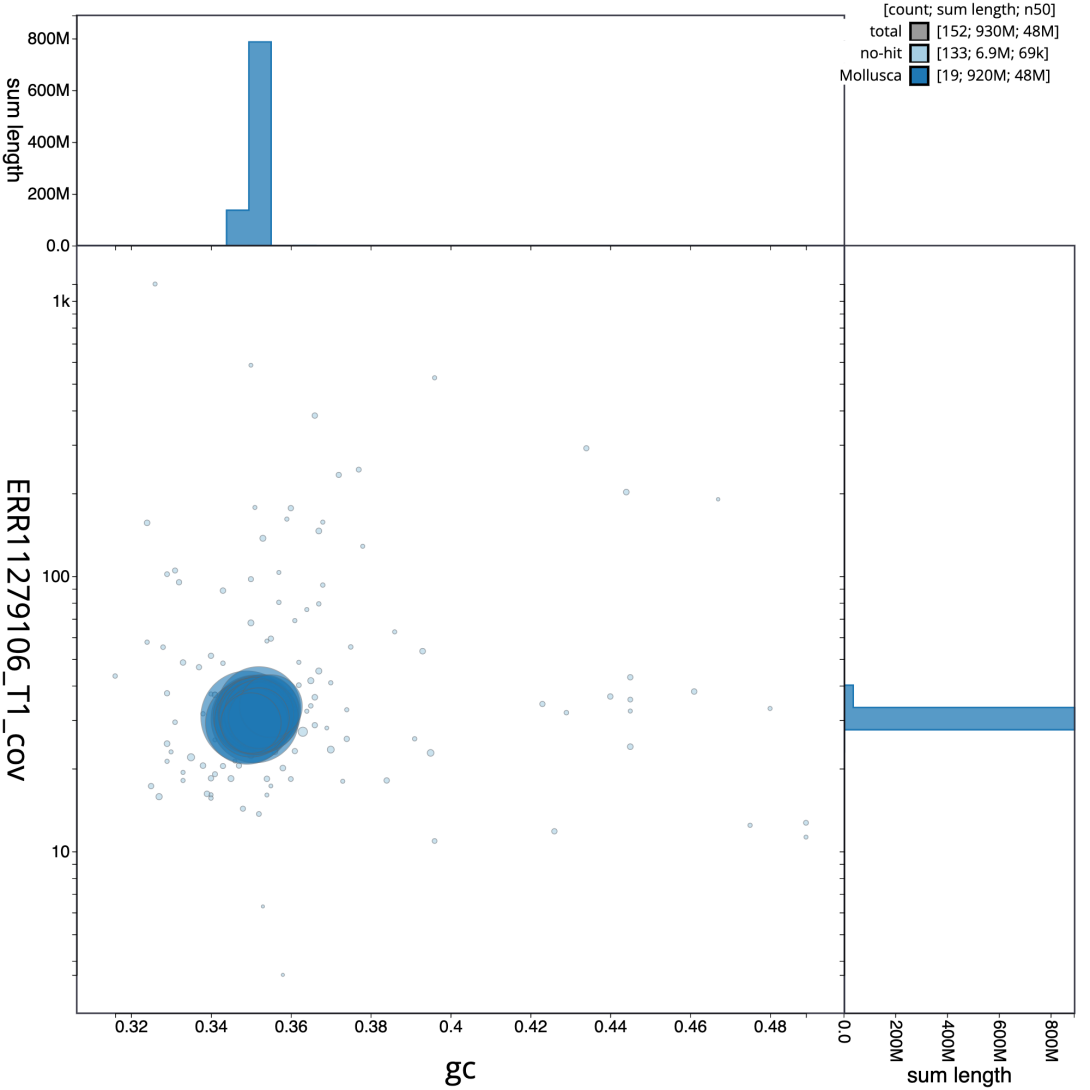
Genome assembly of *Spisula subtruncata*, xbSpiSubt1.1: BlobToolKit GC-coverage plot. Sequences are coloured by phylum. Circles are sized in proportion to sequence length. Histograms show the distribution of sequence length sum along each axis. An interactive version of this figure is available at https://blobtoolkit.genomehubs.org/view/Spisula_subtruncata/dataset/GCA_963678985.1/blob.

**Figure 4.**
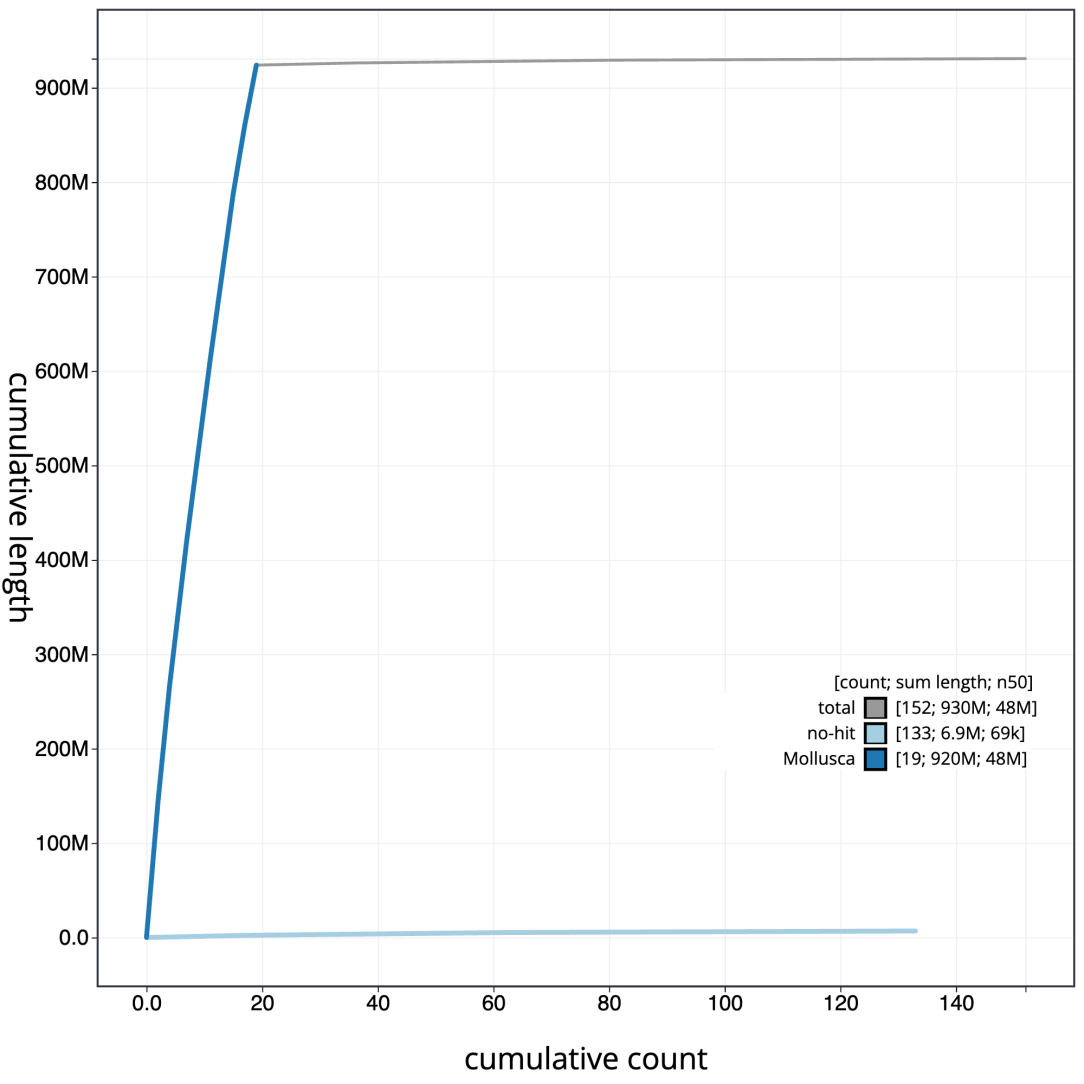
Genome assembly of *Spisula subtruncata*, xbSpiSubt1.1: BlobToolKit cumulative sequence plot. The grey line shows cumulative length for all sequences. Coloured lines show cumulative lengths of sequences assigned to each phylum using the buscogenes taxrule. An interactive version of this figure is available at https://blobtoolkit.genomehubs.org/view/Spisula_subtruncata/dataset/GCA_963678985.1/cumulative.

**Figure 5.**
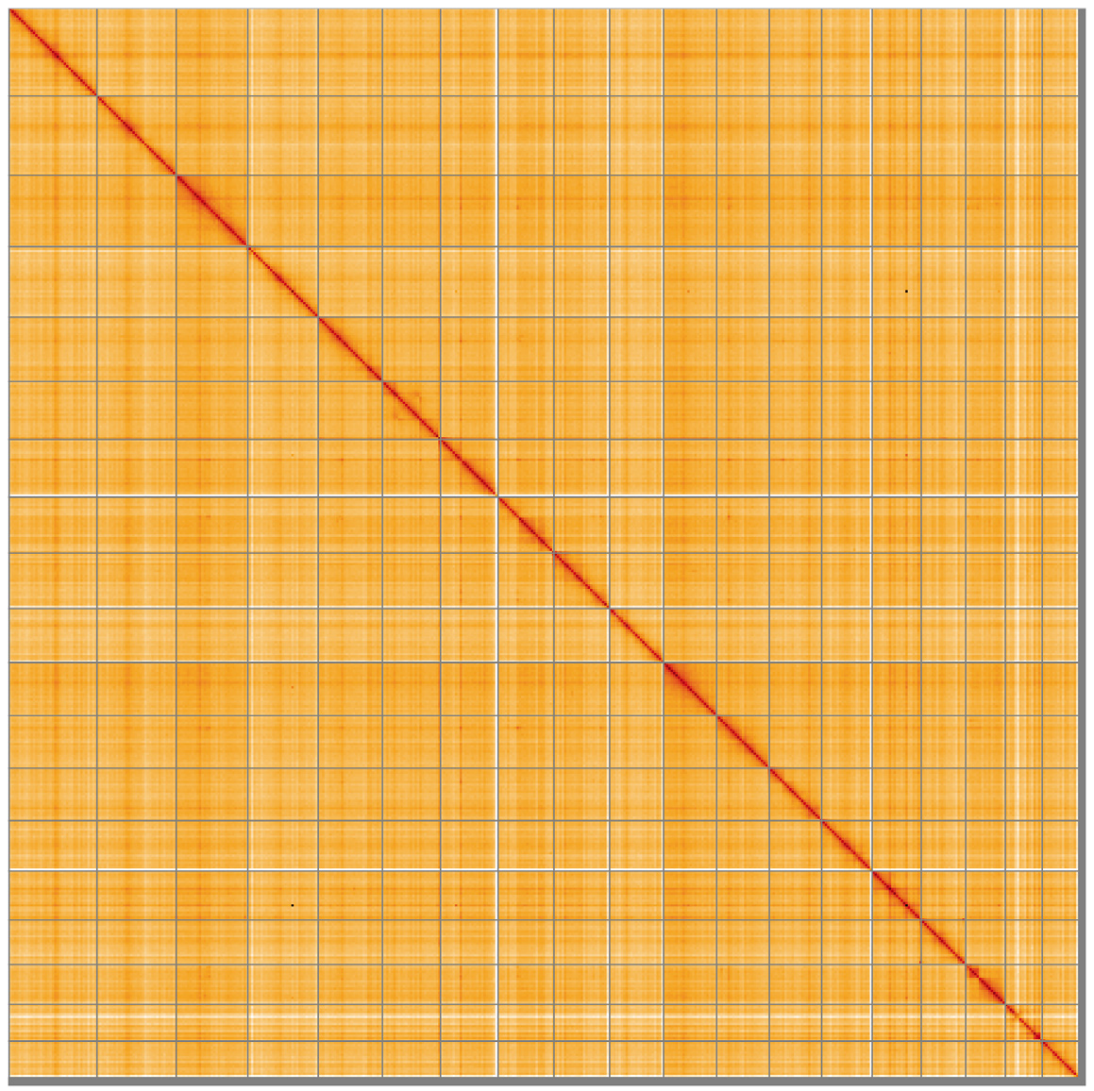
Genome assembly of *Spisula subtruncata*, xbSpiSubt1.1: Hi-C contact map of the xbSpiSubt1.1 assembly, visualised using HiGlass. Chromosomes are shown in order of size from left to right and top to bottom. An interactive version of this figure may be viewed at https://genome-note-higlass.tol.sanger.ac.uk/l/?d=NB6vXH6NQ-m3FVQdho4_Xg.

The final assembly has a total length of 930.8 Mb in 151 sequence scaffolds with a scaffold N50 of 48.3 Mb ( Table 1). The snail plot in Figure 2 provides a summary of the assembly statistics, while the distribution of assembly scaffolds on GC proportion and coverage is shown in Figure 3. The cumulative assembly plot in Figure 4 shows curves for subsets of scaffolds assigned to different phyla. Most (99.25%) of the assembly sequence was assigned to 19 chromosomal-level scaffolds. Chromosome-scale scaffolds confirmed by the Hi-C data are named in order of size ( Figure 5; Table 2). While not fully phased, the assembly deposited is of one haplotype. Contigs corresponding to the second haplotype have also been deposited. The mitochondrial genome was also assembled and can be found as a contig within the multifasta file of the genome submission.

**Table 1.** Genome data for *Spisula subtruncata*, xbSpiSubt1.1.

Project accession data		
Assembly identifier	xbSpiSubt1.1	
Species	*Spisula subtruncata*	
Specimen	xbSpiSubt1	
NCBI taxonomy ID	31202	
BioProject	PRJEB61702	
BioSample ID	SAMEA110450095	
Isolate information	xbSpiSubt1 (PacBio DNA sequencing) xbSpiSubt5 (Illumina Hi-C sequencing) xbSpiSubt10 (Illumina RNA sequencing)	
Assembly metrics *		*Benchmark*
Consensus quality (QV)	67.8	*≥ 50*
*k*-mer completeness	100.0%	*≥ 95%*
BUSCO **	C:80.0%[S:78.5%,D:1.5%], F:4.3%,M:15.7%,n:5,295	*C ≥ 95%*
Percentage of assembly mapped to chromosomes	99.25%	*≥ 95%*
Sex chromosomes	None	*localised * *homologous pairs*
Organelles	Mitochondrial genome: 19.64 kb	*complete single * *alleles*
Raw data accessions		
PacificBiosciences Sequel IIe	ERR11279106	
Hi-C Illumina	ERR11439628	
PolyA RNA-Seq Illumina	ERR11439629	
Genome assembly		
Assembly accession	GCA_963678985.1	
*Accession of alternate haplotype*	GCA_963678955.1	
Span (Mb)	930.8	
Number of contigs	590	
Contig N50 length (Mb)	4.0	
Number of scaffolds	151	
Scaffold N50 length (Mb)	48.3	
Longest scaffold (Mb)	75.87	

* Assembly metric benchmarks are adapted from column VGP-2020 of “Table 1: Proposed standards and metrics for defining genome assembly quality” from Rhie *et al.* (2021). ** BUSCO scores based on the mollusca_odb10 BUSCO set using version v5.4.3. C = complete [S = single copy, D = duplicated], F = fragmented, M = missing, n = number of orthologues in comparison. A full set of BUSCO scores is available at https://blobtoolkit.genomehubs.org/view/Spisula_subtruncata/dataset/GCA_963678985.1/busco.

**Table 2.** Chromosomal pseudomolecules in the genome assembly of *Spisula subtruncata*, xbSpiSubt1.

INSDC accession	Chromosome	Length (Mb)	GC%
OY787660.1	1	75.87	35.0
OY787661.1	2	68.49	35.0
OY787662.1	3	61.79	35.0
OY787663.1	4	60.84	35.0
OY787664.1	5	55.44	35.0
OY787665.1	6	50.23	35.0
OY787666.1	7	49.7	35.5
OY787667.1	8	48.29	35.0
OY787668.1	9	48.08	35.0
OY787669.1	10	46.58	35.0
OY787670.1	11	45.83	35.5
OY787671.1	12	45.54	35.0
OY787672.1	13	45.21	35.0
OY787673.1	14	43.61	35.5
OY787674.1	15	42.4	35.0
OY787675.1	16	38.37	35.0
OY787676.1	17	34.26	35.5
OY787677.1	18	31.85	35.0
OY787678.1	19	31.6	35.0
OY787679.1	MT	0.02	39.5

The estimated Quality Value (QV) of the final assembly is 67.8 with *k*-mer completeness of 100.0%, and the assembly has a BUSCO v completeness of 80.0% (single = 78.5%, duplicated = 1.5%), using the mollusca_odb10 reference set ( *n* = 5,295).

Metadata for specimens, BOLD barcode results, spectra estimates, sequencing runs, contaminants and pre-curation assembly statistics are given at https://links.tol.sanger.ac.uk/species/31202.

## Methods

### Sample acquisition and nucleic acid extraction

A *Spisula subtruncata* (specimen ID MBA-211006-016A, ToLID xbSpiSubt1) was collected from Drakes Island East, Plymouth Sound, Devon, UK (latitude 50.35, longitude –4.15) on 2021-10-06 using a Van Veen grab (RV Sepia). The specimen was collected by Patrick Adkins (Marine Biological Association) and Andrew Mackie (Amgueddfa Cymru) and identified by Anna Holmes (Amgueddfa Cymru) and preserved in liquid nitrogen.

The specimens used for Hi-C sequencing (specimen ID MBA-211008-004E, ToLID xbSpiSubt5) and RNA sequencing (specimen ID MBA-211008-004J, ToLID xbSpiSubt10) were collected from Cawsand Bay, Devon, UK (latitude 50.33, longitude –4.19) on 2021-10-08 using a Van Veen grab (RV Sepia). The specimens were collected by Teresa Darbyshire and Anna Holmes (both Amgueddfa Cymru) and identified by Anna Holmes and preserved in liquid nitrogen.

The workflow for high molecular weight (HMW) DNA extraction at the Wellcome Sanger Institute (WSI) Tree of Life Core Laboratory includes a sequence of core procedures: sample preparation; sample homogenisation, DNA extraction, fragmentation, and clean-up. In sample preparation at the WSI Tree of Life Core Laboratory, the xbSpiSubt1 sample was weighed and dissected on dry ice ( Jay *et al.*, 2023). Somatic tissue was homogenised using a PowerMasher II tissue disruptor ( Denton *et al.*, 2023a).

HMW DNA was extracted in the WSI Scientific Operations core using the Automated MagAttract v2 protocol ( Oatley *et al.*, 2023). The DNA was sheared into an average fragment size of 12–20 kb in a Megaruptor 3 system with speed setting 31 ( Bates *et al.*, 2023). Sheared DNA was purified by solid-phase reversible immobilisation ( Strickland *et al.*, 2023): in brief, the method employs a 1.8X ratio of AMPure PB beads to sample to eliminate shorter fragments and concentrate the DNA. The concentration of the sheared and purified DNA was assessed using a Nanodrop spectrophotometer and Qubit Fluorometer and Qubit dsDNA High Sensitivity Assay kit. Fragment size distribution was evaluated by running the sample on the FemtoPulse system.

RNA was extracted from tissue of xbSpiSubt10 in the Tree of Life Laboratory at the WSI using the RNA Extraction: Automated MagMax™ *mir*Vana protocol ( do Amaral *et al.*, 2023). The RNA concentration was assessed using a Nanodrop spectrophotometer and a Qubit Fluorometer using the Qubit RNA Broad-Range Assay kit. Analysis of the integrity of the RNA was done using the Agilent RNA 6000 Pico Kit and Eukaryotic Total RNA assay.

Protocols developed by the WSI Tree of Life laboratory are publicly available on protocols.io ( Denton *et al.*, 2023b).

### Sequencing

Pacific Biosciences HiFi circular consensus DNA sequencing libraries were constructed according to the manufacturers’ instructions. Poly(A) RNA-Seq libraries were constructed using the NEB Ultra II RNA Library Prep kit. DNA and RNA sequencing was performed by the Scientific Operations core at the WSI on Pacific Biosciences Sequel IIe (HiFi) and Illumina NovaSeq 6000 (RNA-Seq) instruments. Hi-C data were also generated from tissue of xbSpiSubt5 using the Arima2 kit and sequenced on the Illumina NovaSeq 6000 instrument.

### Genome assembly and curation

Assembly was carried out with Hifiasm ( Cheng *et al.*, 2021) and haplotypic duplication was identified and removed with purge_dups ( Guan *et al.*, 2020). The assembly was then scaffolded with Hi-C data ( Rao *et al.*, 2014) using YaHS ( Zhou *et al.*, 2023). The assembly was checked for contamination and corrected using the TreeVal pipeline ( Pointon *et al.*, 2023). Manual curation was performed using JBrowse2 ( Diesh *et al.*, 2023), HiGlass ( Kerpedjiev *et al.*, 2018) and PretextView ( Harry, 2022). The mitochondrial genome was assembled using MitoHiFi ( Uliano-Silva *et al.*, 2023), which runs MitoFinder ( Allio *et al.*, 2020) or MITOS ( Bernt *et al.*, 2013) and uses these annotations to select the final mitochondrial contig and to ensure the general quality of the sequence.

### Final assembly evaluation

The final assembly was post-processed and evaluated with the three Nextflow ( Di Tommaso *et al.*, 2017) DSL2 pipelines “sanger-tol/readmapping” ( Surana *et al.*, 2023a), “sanger-tol/genomenote” ( Surana *et al.*, 2023b), and “sanger-tol/blobtoolkit” ( Muffato *et al.*, 2024). The pipeline sanger-tol/readmapping aligns the Hi-C reads with bwa-mem2 ( Vasimuddin *et al.*, 2019) and combines the alignment files with SAMtools ( Danecek *et al.*, 2021). The sanger-tol/genomenote pipeline transforms the Hi-C alignments into a contact map with BEDTools ( Quinlan & Hall, 2010) and the Cooler tool suite ( Abdennur & Mirny, 2020), which is then visualised with HiGlass ( Kerpedjiev *et al.*, 2018). It also provides statistics about the assembly with the NCBI datasets ( Sayers *et al.*, 2024) report, computes *k*-mer completeness and QV consensus quality values with FastK and MerquryFK, and a completeness assessment with BUSCO ( Manni *et al.*, 2021).

The sanger-tol/blobtoolkit pipeline is a Nextflow port of the previous Snakemake Blobtoolkit pipeline ( Challis *et al.*, 2020). It aligns the PacBio reads with SAMtools and minimap2 ( Li, 2018) and generates coverage tracks for regions of fixed size. In parallel, it queries the GoaT database ( Challis *et al.*, 2023) to identify all matching BUSCO lineages to run BUSCO ( Manni *et al.*, 2021). For the three domain-level BUSCO lineage, the pipeline aligns the BUSCO genes to the Uniprot Reference Proteomes database ( Bateman *et al.*, 2023) with DIAMOND ( Buchfink *et al.*, 2021) blastp. The genome is also split into chunks according to the density of the BUSCO genes from the closest taxonomically lineage, and each chunk is aligned to the Uniprot Reference Proteomes database with DIAMOND blastx. Genome sequences that have no hit are then chunked with seqtk and aligned to the NT database with blastn ( Altschul *et al.*, 1990). All those outputs are combined with the blobtools suite into a blobdir for visualisation.

All three pipelines were developed using the nf-core tooling ( Ewels *et al.*, 2020), use MultiQC ( Ewels *et al.*, 2016), and make extensive use of the Conda package manager, the Bioconda initiative ( Grüning *et al.*, 2018), the Biocontainers infrastructure ( da Veiga Leprevost *et al.*, 2017), and the Docker ( Merkel, 2014) and Singularity ( Kurtzer *et al.*, 2017) containerisation solutions.

Table 3 contains a list of relevant software tool versions and sources.

**Table 3.** Software tools: versions and sources.

Software tool	Version	Source
BEDTools	2.30.0	https://github.com/arq5x/bedtools2
Blast	2.14.0	ftp://ftp.ncbi.nlm.nih.gov/blast/ executables/blast+/
BlobToolKit	4.3.7	https://github.com/blobtoolkit/blobtoolkit
BUSCO	5.4.3	https://gitlab.com/ezlab/busco
BUSCO	5.4.3 and 5.5.0	https://gitlab.com/ezlab/busco
bwa-mem2	2.2.1	https://github.com/bwa-mem2/bwa-mem2
Cooler	0.8.11	https://github.com/open2c/cooler
DIAMOND	2.1.8	https://github.com/bbuchfink/diamond
fasta_ windows	0.2.4	https://github.com/tolkit/fasta_windows
FastK	427104ea91c78c3b8b8b49f1a7d6bbeaa869ba1c	https://github.com/thegenemyers/FASTK
GoaT CLI	0.2.5	https://github.com/genomehubs/goat-cli
Hifiasm	0.16.1-r375	https://github.com/chhylp123/hifiasm
HiGlass	1.11.6	https://github.com/higlass/higlass
HiGlass	44086069ee7d4d3f6f3f0012569789ec138f42b84 aa44357826c0b6753eb28de	https://github.com/higlass/higlass
MerquryFK	d00d98157618f4e8d1a9190026b19b471055b22e	https://github.com/thegenemyers/ MERQURY.FK
MitoHiFi	2	https://github.com/marcelauliano/ MitoHiFi
MultiQC	1.14, 1.17, and 1.18	https://github.com/MultiQC/MultiQC
NCBI Datasets	15.12.0	https://github.com/ncbi/datasets
Nextflow	23.04.0-5857	https://github.com/nextflow-io/nextflow
PretextView	0.2	https://github.com/wtsi-hpag/PretextView
purge_dups	1.2.3	https://github.com/dfguan/purge_dups
samtools	1.16.1, 1.17, and 1.18	https://github.com/samtools/samtools
sanger-tol/ genomenote	1.1.1	https://github.com/sanger-tol/ genomenote
sanger-tol/ readmapping	1.2.1	https://github.com/sanger-tol/ readmapping
Seqtk	1.3	https://github.com/lh3/seqtk
Singularity	3.9.0	https://github.com/sylabs/singularity
TreeVal	1.0.0	https://github.com/sanger-tol/treeval
YaHS	yahs-1.1.91eebc2	https://github.com/c-zhou/yahs

### Wellcome Sanger Institute – Legal and Governance

The materials that have contributed to this genome note have been supplied by a Darwin Tree of Life Partner. The submission of materials by a Darwin Tree of Life Partner is subject to the **‘Darwin Tree of Life Project Sampling Code of Practice’**, which can be found in full on the Darwin Tree of Life website here. By agreeing with and signing up to the Sampling Code of Practice, the Darwin Tree of Life Partner agrees they will meet the legal and ethical requirements and standards set out within this document in respect of all samples acquired for, and supplied to, the Darwin Tree of Life Project.

Further, the Wellcome Sanger Institute employs a process whereby due diligence is carried out proportionate to the nature of the materials themselves, and the circumstances under which they have been/are to be collected and provided for use. The purpose of this is to address and mitigate any potential legal and/or ethical implications of receipt and use of the materials as part of the research project, and to ensure that in doing so we align with best practice wherever possible. The overarching areas of consideration are:

•     Ethical review of provenance and sourcing of the material

•     Legality of collection, transfer and use (national and international)

Each transfer of samples is further undertaken according to a Research Collaboration Agreement or Material Transfer Agreement entered into by the Darwin Tree of Life Partner, Genome Research Limited (operating as the Wellcome Sanger Institute), and in some circumstances other Darwin Tree of Life collaborators.

## Data Availability

European Nucleotide Archive: *Spisula subtruncata* (cut surfclam). Accession number PRJEB61702; https://identifiers.org/ena.embl/PRJEB61702 ( Wellcome Sanger Institute, 2023). The genome sequence is released openly for reuse. The *Spisula subtruncata* genome sequencing initiative is part of the Darwin Tree of Life (DToL) project. All raw sequence data and the assembly have been deposited in INSDC databases. The genome will be annotated using available RNA-Seq data and presented through the Ensembl pipeline at the European Bioinformatics Institute. Raw data and assembly accession identifiers are reported in Table 1.

## Author information

Members of the Marine Biological Association Genome Acquisition Lab are listed here: https://doi.org/10.5281/zenodo.8382513.

Members of the Darwin Tree of Life Barcoding collective are listed here: https://doi.org/10.5281/zenodo.4893703.

Members of the Wellcome Sanger Institute Tree of Life Management, Samples and Laboratory team are listed here: https://doi.org/10.5281/zenodo.10066175.

Members of Wellcome Sanger Institute Scientific Operations: Sequencing Operations are listed here: https://doi.org/10.5281/zenodo.10043364.

Members of the Wellcome Sanger Institute Tree of Life Core Informatics team are listed here: https://doi.org/10.5281/zenodo.10066637.

Members of the Tree of Life Core Informatics collective are listed here: https://doi.org/10.5281/zenodo.5013541.

Members of the Darwin Tree of Life Consortium are listed here: https://doi.org/10.5281/zenodo.4783558.
