# Supplementary material for: The genome sequence of the cut surfclam, Spisula subtruncata (da Costa, 1778)
Source: Wellcome Open Res. 2024 May 21;9:286. [Version 1] doi: 10.12688/wellcomeopenres.22286.1 (PMC11249531; doi:10.12688/wellcomeopenres.22286.1)
Supplement: Peer review history

# Reviewer report: Reviewer response for version 1

**DOI:** 10.21956/wellcomeopenres.24563.r87881

**Published:** 2024-08-13

**Authors:** Zhihua Lin, Yongbo Bao

**Affiliations:** 1 Zhejiang Wanli University, Ningbo, Zhejiang, China

This Data note provides a necessary introduction to the species cut surfclam, *Spisula subtruncata*. The methods for genome sequencing and analysis are reasonable, and the description is detailed. The data have been registered in the European Nucleotide Archive, making it easily accessible to readers. Uploading the data to NCBI simultaneously can allow more readers to access it. In conclusion, I believe this paper can be indexed.

Are sufficient details of methods and materials provided to allow replication by others?

Yes

Is the rationale for creating the dataset(s) clearly described?

Yes

Are the datasets clearly presented in a useable and accessible format?

Yes

Are the protocols appropriate and is the work technically sound?

Yes

Reviewer Expertise:

Immunogenetics.

We confirm that we have read this submission and believe that we have an appropriate level of expertise to confirm that it is of an acceptable scientific standard.

# Reviewer report: Reviewer response for version 1

**DOI:** 10.21956/wellcomeopenres.24563.r87885

**Published:** 2024-07-15

**Authors:** Daniel Garcia-Souto

**Affiliations:** 1 University of Santiago de Compostela, Santiago de Compostela, Galicia, Spain

Adkins et al. present the first comprehensive description of the genome of the surfclam Spisula subtruncata. The assembled genome demonstrates high-quality continuity; however, its completeness appears to be somewhat limited, with a BUSCO completeness score of 80%. This is relatively low compared to similar genomes produced using the same methodology and falls below the desired standards. Nonetheless, this genome represents a valuable addition to the databases for an underrepresented species and deserves recognition in this manuscript.

 The lower completeness score might be attributed to challenges associated with repetitive DNA sequences, as Spisula subtruncata is known to contain a significant amount of satellite DNA, predominantly within the heterochromatic regions of its chromosomes. This newly assembled genome will facilitate further investigations by other researchers into these repetitive elements and their genomic implications.

Are sufficient details of methods and materials provided to allow replication by others?

Yes

Is the rationale for creating the dataset(s) clearly described?

Yes

Are the datasets clearly presented in a useable and accessible format?

Yes

Are the protocols appropriate and is the work technically sound?

Yes

Reviewer Expertise:

Molecular cytogenetics, malacology, genomics, repetitive DNA

I confirm that I have read this submission and believe that I have an appropriate level of expertise to confirm that it is of an acceptable scientific standard.

## References

[1] : Methylation profile of a satellite DNA constituting the intercalary G+C-rich heterochromatin of the cut trough shell Spisula subtruncata (Bivalvia, Mactridae). *Sci Rep* .2017;7(1) : 10.1038/s41598-017-07231-7 6930 10.1038/s41598-017-07231-7 28761142 PMC5537241

# Reviewer report: Reviewer response for version 1

**DOI:** 10.21956/wellcomeopenres.24563.r85482

**Published:** 2024-06-10

**Authors:** Ruiqi Li

**Affiliations:** 1 University of Colorado Boulder, Boulder, Colorado, USA

Adkins et al. presented a high-quality genome of *Spisula subtruncata*, which will be valuable for future studies of this economic-important species. I have a few minor comments:

 1. Introduction: The authors mention two forms of *S. subtruncata*. They should explain whether these forms have any genetic differences and specify which form was sequenced.

 2. Figure 1: Include a scale bar in the specimen photo. If a scale bar is unavailable, state the adult size in the figure caption.

 3. BUSCO Score: The BUSCO score appears low. Can the authors provide potential reasons, such as the nature of the genome, assembly/sequencing issues, or other factors?

Are sufficient details of methods and materials provided to allow replication by others?

Yes

Is the rationale for creating the dataset(s) clearly described?

Yes

Are the datasets clearly presented in a useable and accessible format?

Yes

Are the protocols appropriate and is the work technically sound?

Yes

Reviewer Expertise:

Genomics

I confirm that I have read this submission and believe that I have an appropriate level of expertise to confirm that it is of an acceptable scientific standard.
